# Transient Lipid-Protein Structures and Selective Ganglioside Uptake During α-Synuclein-Lipid Co-aggregation

**DOI:** 10.3389/fcell.2021.622764

**Published:** 2021-02-18

**Authors:** Ricardo Gaspar, Ilaria Idini, Göran Carlström, Sara Linse, Emma Sparr

**Affiliations:** ^1^Division of Physical Chemistry, Department of Chemistry, Lund University, Lund, Sweden; ^2^Division of Biochemistry and Structural Biology, Department of Chemistry, Lund University, Lund, Sweden; ^3^Centre for Analysis and Synthesis, Department of Chemistry, Lund University, Lund, Sweden

**Keywords:** amyloid formation, lipid-protein co-assembly, lipid selectivity, cryo-EM, NMR

## Abstract

α-Synuclein is a membrane-interacting protein involved in Parkinson’s disease. Here we have investigated the co-association of α-synuclein and lipids from ganglioside-containing model membranes. Our study relies on the reported importance of ganglioside lipids, which are found in high amounts in neurons and exosomes, on cell-to-cell prion-like transmission of misfolded α-synuclein. Samples taken along various stages of the aggregation process were imaged using cryogenic transmission electron microscopy, and the composition of samples corresponding to the final state analyzed using NMR spectroscopy. The combined data shows that α-synuclein co-assembles with lipids from the ganglioside (GM1)-containing model membranes. The lipid-protein samples observed during the aggregation process contain non-vesicular objects not present at the final stage, thus capturing the co-existence of species under non-equilibrium conditions. A range of different lipid-protein co-assemblies are observed during the time course of the reaction and some of these appear to be transient assemblies that evolve into other co-aggregates over time. At the end of the aggregation reaction, the samples become more homogeneous, showing thin fibrillar structures heavily decorated with small vesicles. From the NMR analysis, we conclude that the ratio of GM1 to phosphatidyl choline (PC) in the supernatant of the co-aggregated samples is significantly reduced compared to the GM1/PC ratio of the lipid dispersion from which these samples were derived. Taken together, this indicates a selective uptake of GM1 into the fibrillar aggregates and removal of GM1-rich objects from the solution.

## Introduction

The deposition of protein-rich aggregates and the spreading of the pathology throughout the brain are hallmarks of Parkinson’s disease (Schulz-Schaeffer, 2010). The aggregates are termed Lewy bodies and Lewy Neurites, and α-synuclein (α-syn) is recognized as their main protein component. The biological function of α-syn is still not fully understood, but due to its location in neurons and its interaction with lipid membranes, it has been suggested to modulate the dopamine neurotransmission by regulating synaptic vesicle trafficking (Burré, 2015). While primary nucleation of pure α-syn is very slow in bulk solution, this event appears to be accelerated by the presence of lipid membranes, which have been found to influence α-syn aggregation process by facilitating heterogeneous primary nucleation (Grey et al., 2015; Gaspar et al., 2018). Furthermore, associated lipids have been detected in amyloid fibrils formed in the presence of lipid membranes *in vivo* as well as *in vitro* (Halliday et al., 2005; Reynolds et al., 2011; Hellstrand et al., 2013; van Maarschalkerweerd et al., 2014). These observations imply that the lipid membrane is not simply a catalyzing surface but lipids are also active players in the aggregation reaction. α-Syn aggregation has been linked to membrane disruption in cells and of model membranes (Davidson et al., 1998; Furukawa et al., 2006; van Rooijen et al., 2009).

While the main focus in amyloid research has been on protein-protein interactions, and on the interaction of the protein (monomers and aggregated forms) with membranes, the uptake of lipid into forming amyloid aggregates is far less understood. The lipid membrane is a self-assembled dynamic structure rather than an intact and inert entity. The amphiphilic lipids can rearrange into new assemblies together with other macromolecules when the solution conditions are changed. Co-assembly of protein and lipids implies extraction of components from the membrane. This likely affects the membrane structure and function, and may have important pathological consequences. The propensity of lipid molecules to be extracted from the lipid membranes to the aggregates will depend on specific protein-lipid interactions, as well as, general physico-chemical properties of the lipids, including solubility in water and the free energy of transfer from the lipid membrane to amyloid aggregate. Molecular features governing solubility and transfer include acyl chain identity as well as headgroup charge and structure. Lipid-protein co-assembly is expected to have large consequences for the physico-chemical properties of the formed aggregates, including their stability and surface properties. Co-aggregation in amyloid formation is thus expected to modulate surface nucleation as well as the interactions with other molecules or cells.

In this paper we study the process of lipid-protein co-assembly in amyloid formation. We follow the structure evolution at distinct stages of the co-assembly process using cryo electron microscopy (cryo-EM). We investigate whether there is selective uptake of certain lipid species into the forming amyloid aggregates using ^1^H NMR spectroscopy. The model systems investigated contain α-syn and ganglioside-containing vesicles. Gangliosides were chosen because such lipids are present at relatively high concentrations in neurons (Palmano et al., 2015) and in exosomes (Grey et al., 2015), which are biological secreted lipid vesicles that have been growingly linked to the spreading of pathological forms of α-syn (Dunning et al., 2012). Exosomes were recently found to accelerate α-syn aggregation and this effect was attributed to the presence of ganglioside lipids (Grey et al., 2015). Ganglioside lipids have been associated with several physiological processes, including cell signaling, neuronal protection, neuronal recovery and apoptosis (Malisan and Testi, 2002; Sonnino et al., 2013; Aureli et al., 2016; Schneider et al., 2019). α-Syn has also been reported to associate with lipid rafts containing GM1 and GM3 (Fortin et al., 2004; Park et al., 2009; Grey et al., 2015; Gaspar et al., 2018). Aggregation of α-syn in the presence of GM1 containing lipid vesicles is strongly dependent on the lipid-to-protein molar ratio. For this particular system and under these experimental conditions the aggregation occurs at a higher rate for L/Ps 50:1 and 25:1 (Supplementary Figure S1).

## Materials and Methods

### GM1-DOPC Vesicle Preparation

Lyophilized GM1 from ovine brain and DOPC (1,2-dioleoyl-*sn*-glycero-3-phosphocoline) were obtained from Avanti Polar Lipids. Lipid stock solutions were prepared in chloroform/methanol 2:1 (v/v), with a thin lipid film obtained after air-drying. To assure the complete solvent evaporation from the lipid films, these were left overnight in a vacuum chamber. The lipid films were then rehydrated with 10 mM MES pH 5.5 buffer. Samples were then vortexed and sonicated for 15 min, 10 s on/off duty at 75% amplitude on ice. The lipid dispersions were centrifuged for 10 min at 13,000 rpm to remove any contaminating particles from the probe sonicator tip. Lipid dispersions were made for mixtures composed of DOPC/GM1 at molar ratios of 9/1 and 7/3.

### α-Syn Expression and Purification

Human α-syn was expressed in *E. coli* and purified using heat treatment, ion exchange and gel-filtration chromatography, as previously described (Davidson et al., 1998). Gel-filtration is a crucial step to isolate pure monomeric α-syn in the desired degassed experimental buffer, 10 mM MES pH 5.5. Protein sample corresponding to the central region of the peak is collected. The peptide concentration was determined by absorbance at 280 nm using an extinction coefficient ε = 5,800 l mol^–1^ cm^–1^.

### Cryo-EM: Sample Preparation and Measurements

Samples for cryo-EM were aliquoted in non-binding PEGylated plates and sealed with a plastic film to avoid evaporation at 37°C under quiescent conditions. The aggregation kinetics of 20 μM α-syn in the presence of 0.5 mM DOPC/GM1 9/1 lipid membranes was monitored for sample repeats supplemented with 20 μM ThT by recording the fluorescence at 480 nm with excitation at 440 nm. At defined time points along the aggregation reaction samples without ThT were retrieved and used immediately for cryo-EM imaging.

Specimens for electron microscopy were prepared in a controlled environment vitrification system (CEVS) to ensure stable temperature and to avoid loss of solution during sample preparation. The specimens were prepared as thin liquid films, <300 nm thick, on lacey carbon filmed copper grids and plunged into liquid ethane at −180°C. This leads to vitrified specimens, avoiding component segmentation and rearrangement, and water crystallization, thereby preserving original microstructures. The vitrified specimens were stored under liquid nitrogen until measured. A Fischione Model 2550 cryo transfer tomography holder was used to transfer the specimen into the electron microscope, JEM 2200FS, equipped with an in-column energy filter (Omega filter), which allows zero-loss imaging. The acceleration voltage was 200 kV and zero-loss images were recorded digitally with a TVIPS F416 camera using SerialEM under low dose conditions with a 30 eV energy selecting slit in place. Experiments were also conducted using another electron microscope. Here, samples stored under liquid nitrogen were transferred using Oxford CT3500 cryoholder and its workstation into the electron microscope, Philips CM120 Biotwin Cryo, equipped with a post-column energy filter, Gatan GIF100. The acceleration voltage was 120 kV and images were recorded digitally with a CCD camera under low electron dose conditions.

### NMR: Sample Preparation and Measurements

Samples for NMR analysis were prepared as follows: α-syn monomer was isolated in 10 mM MES buffer pH 5.5 using a 24 mL Superdex75 column (GE healthcare) followed by desalting in pure water (MilliQ) using a HiTrap Desalting 5 mL column prepacked with Sephadex G-25 Superfine resin (GE Healthcare). The monomeric protein was then mixed with the lipid dispersion at lipid-to-protein molar ratio of 6/1 (protein concentration 100 μM) and 10/1 (protein concentration 140 μM), and was left to aggregate under stirring in a low-binding Eppendorf tube during 72 h at 37°C. Samples were then centrifuged at 10,000 rpm for 2 min. The supernatant was separated from the sedimented fibrils. In order to avoid contamination of the supernatant sample with fibril fragments, only the upper part of the supernatant (ca. half of the total supernatant volume) was used. The supernatant and fibril samples were freeze-dried and re-suspended in deuterated chloroform-methanol (2:1).

One-dimensional (1D) ^1^H NMR spectra at 25°C were acquired on an Agilent 600 MHz VNMRS spectrometer equipped with an inverse HCN probe. A total of 576–4,096 scans were acquired using a 30° pulse of length 2.9 μs, an acquisition time of 1 s, and an additional relaxation delay (d1) of 2 s. The data were Fourier transformed into 64 k data points using an exponential window function of 1 Hz, and the relevant peaks were integrated after careful base line correction. The samples contained a small amount of tetramethylsilane (TMS), which was used for referencing.

## Results and Discussion

**FIGURE 1:**
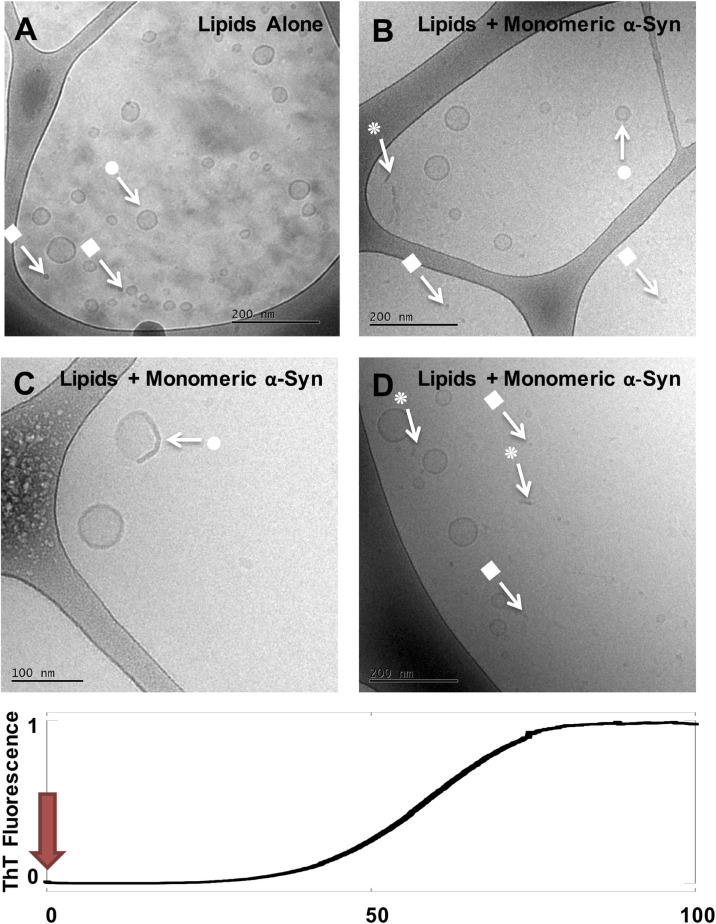
Cryo-EM imaging of DOPC/GM1 lipid dispersion in the absence and presence of monomeric α-syn. **(A)** 1 mM DOPC/GM1 9/1 lipid dispersion. **(B–D)** Three example images for the same sample: 20 μM monomeric α-syn added to 0.5 mM DOPC/GM1 9/1, *t* = 0. The sample was frozen and imaged immediately after the protein was added. Arrows with • symbols indicate examples of lipid vesicles, ■ micelles and 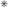 rod-like or edge-on lipid disc-like objects.

**FIGURE 2:**
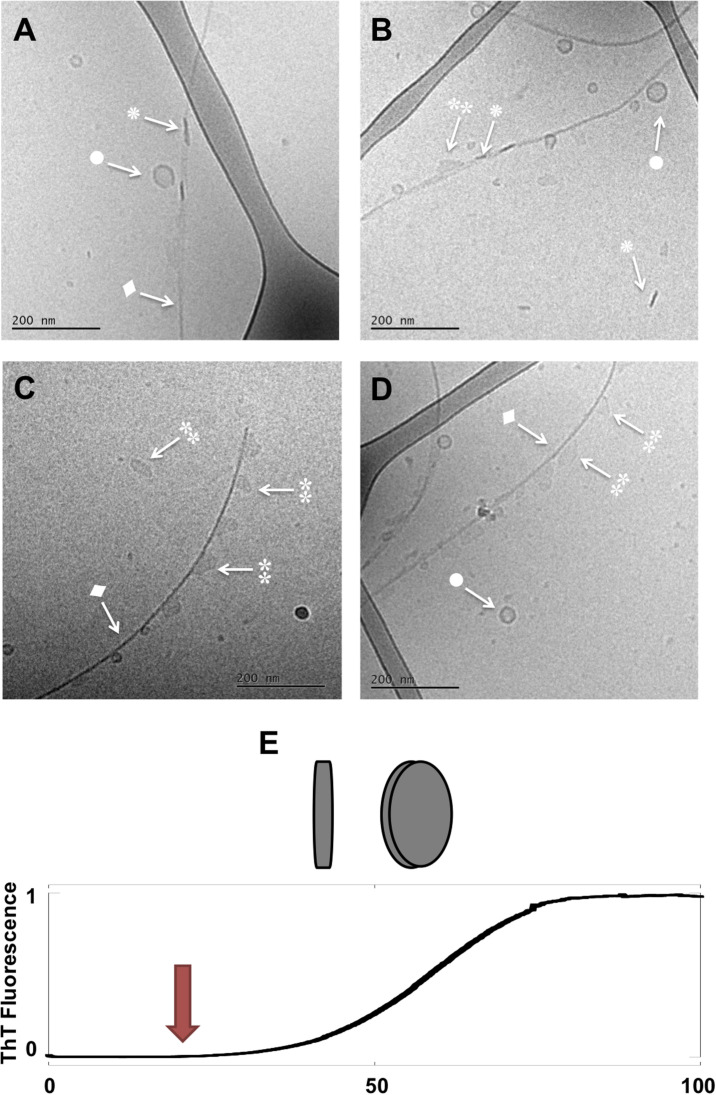
Lipid-protein samples at the middle of the lag phase (*t* = 14 h). **(A–D)** Four example images for the same sample: 0.5 mM lipids (DOPC/GM1 molar ratio 9/1) left aggregating in the presence of 20 μM α-syn. Arrows with • symbols indicate examples of lipid vesicles, ◆ fibrillar structures, 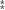 irregular objects and 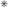 rod-like objects that can be interpreted as edge-on lipid discs. **(E)** Schematic representation of lipodiscs seen from different angles that might correspond to 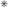 and 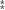 in the cryo-TEM images.

**FIGURE 3:**
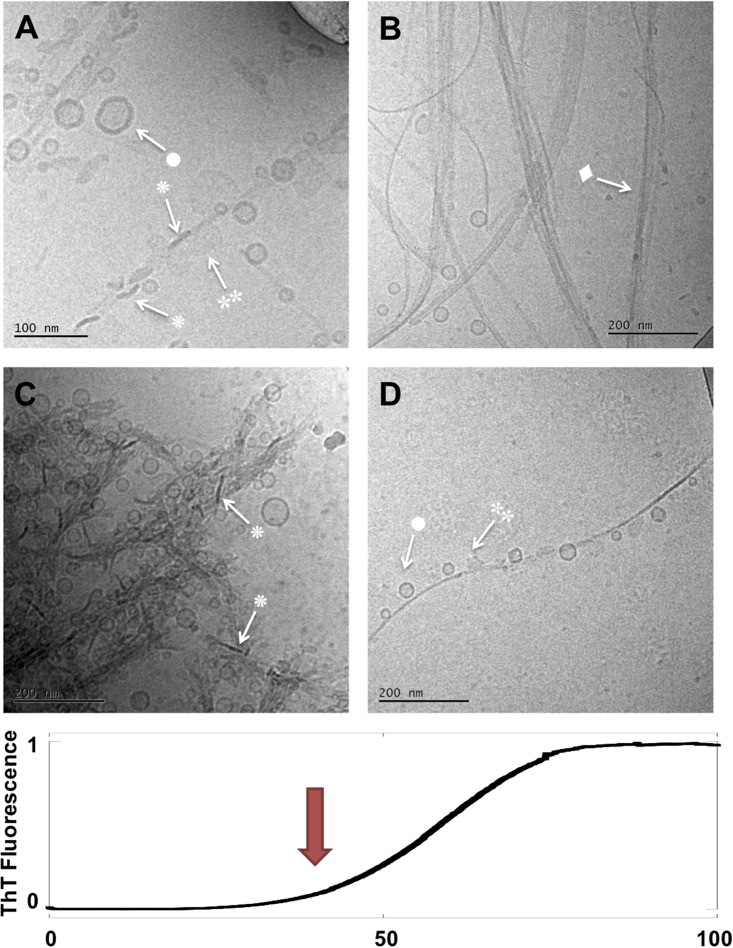
Lipid-protein samples at the end of the lag phase and at the start of the macroscopic transition (*t* = 40 h). **(A–D)** Four example images for the same sample: 0.5 mM lipids (DOPC/GM1 molar ratio 9/1) left aggregating in the presence of 20 μM α-syn. Arrows with • symbols indicate examples of lipid vesicles, ◆ fibrillar structures, 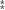 irregular objects and ◆ rod-like objects that can be interpreted as edge-on lipid discs.

**FIGURE 4:**
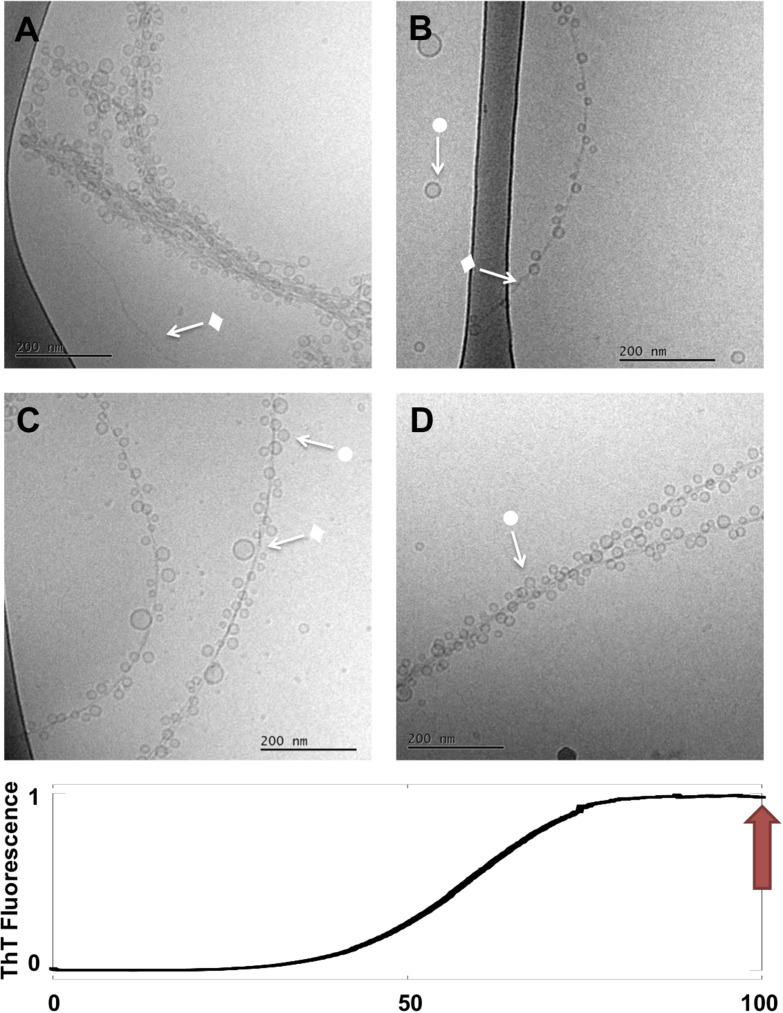
Lipid-protein samples at the end of the reaction (*t* = 90 h). **(A–D)** Four example images for the same sample: 0.5 mM lipids (DOPC/GM1 molar ratio 9/1) left aggregating in the presence of 20 μM α-syn. Arrows with • symbols indicate examples of lipid vesicles and ◆ fibrillar structures. No rod-like or disc-like objects are observed.

**FIGURE 5:**
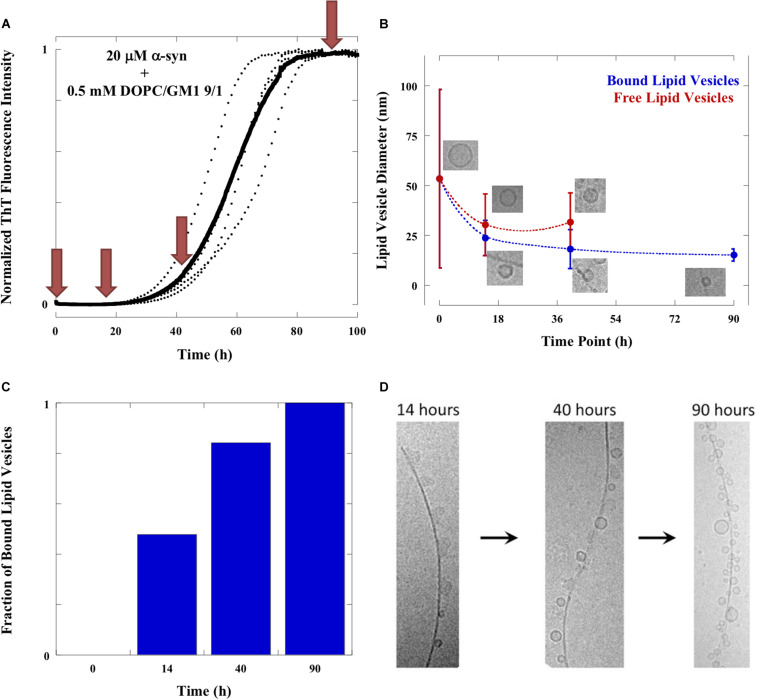
Evolution of samples. **(A)** Aggregation of 20 μM α-syn in the presence of 0.5 mM DOPC/GM1 9/1 lipid vesicles monitored by ThT fluorescence at 37°C under quiescent conditions. The average trace is shown in bold with experimental repeats as dotted lines. Red arrows indicate the time points at which samples were imaged with cryo-EM. **(B)** Sizes of lipid vesicles bound to the fibrils (blue line) and free in solution (red line) as a function of time along the aggregation reaction. The values shown are mean ± SD. The number of free lipid vesicles at time point 90 h is too low to get good statistics and therefore not included in the figure. The number of free and bound lipid vesicles were counted from at least 10 frames of the cryo-EM images. **(C)** The number of bound lipid vesicles in relation to total number of lipid vesicles as a function of time. **(D)** Time evolution of fibrils adsorbed objects. The fraction of irregular object is high at 14 h, intermediate at 40 h and negligible at 90 h. The fraction of small vesicles is low at 14 h, intermediate at 40 h and high at 90 h.

We have in previous studies identified experimental conditions leading to reproducible α-syn aggregation kinetics (Buell et al., 2014; Grey et al., 2015; Gaspar et al., 2017; Figure 5A), making it possible to collect samples at defined stages along the aggregation process for cryo-EM imaging. In the present experiments, monomeric α-syn was mixed with lipid vesicles (50–100 nm in diameter) composed of DOPC and GM1 (molar ratio PC/GM1 9/1) at a lipid-to-protein (L/P) molar ratio of 25/1 at mildly acidic pH. Four different time points were chosen to represent various stages of the macroscopically observable aggregation process under quiescent conditions as monitored by ThT fluorescence: directly after mixture of α-syn and GM1 lipid vesicles (*t* = 0), in the middle of the lag phase (*t* = 14 h), at the end of the lag-phase when ca. 10% of the final ThT intensity is reached (*t* = 40 h), and at the plateau of the ThT kinetic trace to represent the final stage of the aggregation process (*t* ≈ 90 h). Two samples were taken at each time point, directly frozen at −180°C in liquid ethane and imaged by cryo-EM on glow-discharged carbon grids (Figures 1–4). As a control, samples of dispersed lipids alone at t = 0 were imaged (Figure 1A). This control sample contains both unilamellar vesicles and small micelles, consistent with previous reports (Mojumbar et al., 2019). Additionally, the effect of mechanical insult on aggregate morphology was investigated, through the imaging of final stage samples produced under quiescent and stirring (200 rpm) conditions, respectively (Supplementary Figure S3). The experiments were performed at mildly acidic pH and low ionic strength (10 mM MES/NaOH buffer pH 5.5). Under these solution conditions, the dominant nucleation aggregation event for α-syn alone is secondary nucleation, which involves nucleation of monomers on the surface of existing fibrils (Buell et al., 2014; Gaspar et al., 2017). Furthermore, membrane-induced aggregation is more efficient at mildly acidic conditions compared to neutral pH, where systematic studies have revealed that only certain lipid systems, such as, lipids with short (C12-C14) saturated acyl chains and PS head groups act as triggers of α-syn aggregation (Galvagnion et al., 2016; Gaspar et al., 2018). In the QCM-D experiments shown in Supplementary Figure S2, we observed distinct differences when adding α-syn to deposited lipid bilayers composed of 9/1 DOPC/GM1 at mildly acidic pH (pH 5.5) and neutral pH. At the lower pH, α-syn shows rather strong adsorption to the bilayers, while at neutral pH there is less detectable protein adsorption to the bilayer with the same composition. The mildly acidic pH has physiological relevance because α-syn is found in some cellular compartments, such as lysosomes and endosomes, with an acidic lumen (Grey et al., 2015).

### Evolution of α-Syn Samples Followed With Cryo-EM Imaging and ThT Fluorescence

Directly after mixing with GM1-containing vesicles, *t* = 0, α-syn appears to associate with the membrane as we observe a distinct change toward irregular/non-spherical shaped vesicles (arrows with • in Figures 1B–D). There are also indications of membrane thickening, or increased membrane contrast to the electrons, in the presence of α-syn. Non-uniform protein binding to the membranes is consistent with previous studies of other anionic model membranes (Grey et al., 2011). Both in the absence (Figure 1A) and presence (Figures 1B–D) of α-syn, the lipid samples show co-existence of vesicles and smaller aggregates interpreted as GM1-rich micelles (arrows with ■). In the protein-containing samples, we also observe rod-like or disc-like objects (arrows with 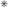 in Figures 1B–D). In the following images (Figures 2–4), we use the same symbols to indicate vesicles (•), small micellar-like aggregates (■), and rod- or disk-like aggregates (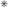). In order to portrait a more representative overall picture, for each sample investigated multiple frames are shown. As will be evident from the comparisons at the different time points, the samples are relatively uniform in the sense of what type of structures that are present for the initial state as well as for the state corresponding to the ThT plateau, while for the intermediate time points the sample is very heterogeneous and contain multiple types of structures.

We propose the following interpretation of the cryo-EM images in Figures 1–4. Immediately after mixing of monomeric α-syn with DOPC/GM1 vesicles, the protein associates with the lipid membranes. This is inferred from the QCM-D data in Supplementary Figure S2 showing protein adsorption. The binding to the GM1-containing membrane is also associated with a conformational change of the α-syn, as previously demonstrated by means of circular dichroism (Gaspar et al., 2018). Protein adsorption may also explain the observations of membrane thickening and vesicle deformation in Figure 1.

Next, structural rearrangement and co-assembly processes take place. This is inferred from the images taken in the middle of the lag-phase showing the co-existence of several different types of co-assemblies (Figure 2, *t* = 14 h). Thin and bent fibrils are decorated with small lipid vesicles (•). Furthermore, the sample contains other less well-defined assemblies of much more irregular shape than the deformed vesicles seen at *t* = 0. These objects appear a bit “blurry” with low and no variations in contrast as seen in the vesicles (indicated with 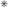

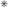
). In addition, there are small objects with high contrast, which look like rods (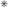).

We note that fibrils (◆) are present in the sample at time points before the observed strong increase in ThT fluorescence. Since elongation and secondary nucleation are fast events compared to primary nucleation, the first fibrils form at the very early stages of the lag phase. The observation of fibrils in the sample taken half-way through the lag phase is thus an expected result in accordance with earlier studies for the amyloid-β peptide (Arosio et al., 2014, 2015). Because of the low signal-to-noise ratio of the ThT fluorescence, our ThT-based assay fails to detect fibrils until their mass concentration amounts to ca. 1% of the total protein. The close to exponential rise in the concentration of fibrils during the lag phase has been determined using more sensitive techniques (Arosio et al., 2014).

In addition to the fibrils, we see a range of smaller structures that are likely rich in lipids. First, we identify free lipid-rich objects that are vesicles. The vesicles likely also contain associated protein (Galvagnion et al., 2016; Gaspar et al., 2018; Iyer and Claessens, 2019). Second, we observe very small vesicles of ca. 20 nm diameter, which are associated with the protein aggregates at the long sides of the fibrils. Third, we see irregular objects of ca. 50 nm in diameter, which are either associated along the sides of the fibrils or free in solution. These objects appear “blurry” with no variations in contrast as seen in vesicles. In the same samples, we see rod-like objects of high contrast, and several of these appear associated with fibrils. The “blurry” and rod-like objects may be distinct structures, but it is possible that they represent the same type of object seen from different angles, e.g., ganglioside-rich lipodiscs (Figure 2E). Such lipodiscs can be stabilized by the ganglioside lipids with large sugar headgroups, and/or by the associated α-syn (Eichmann et al., 2016; Mojumbar et al., 2019). Lipodiscs have indeed been detected for the lipid model with large hydrophilic headgroups (Sandström et al., 2008; Zetterberg et al., 2016), as well as, for α-syn mixed with anionic phospholipids (Eichmann et al., 2016). The objects that decorate the fibrils appear in many places at similar distance from each other, such that there are stretches of fibrils that appear devoid of any obvious lipid particle, including vesicles, lipodiscs or irregular objects.

Moving to the next time point (40 h) we can observe that the sample has clearly evolved during the second half of the lag phase and into the early part of the macroscopically observable transition in the ThT curve (Figure 3). The same four types of objects identified after 14 h are still observed, but their relative abundance seems to have changed. For example, the more isolated and decorated fibrils, seem to carry a larger number of small vesicles (•) and a smaller number of irregular blurry (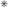

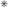
) and rod-like objects (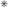) associated along the sides of these fibrils. Another sign of evolution of the sample is the apparent clustering of fibrils in lateral assemblies or in networks that seem to enclose vesicles.

Further evolution of the co-aggregates can be deduced from the last stage images (t ≈ 90 h), in which much of the heterogeneity has disappeared. Images taken at the end of the aggregation reaction reveal thin fibrils heavily decorated with very small and relatively monodisperse vesicles along their entire length (•) (Figure 4). Only very few free vesicles can be seen. Rod-like and “blurry” objects are no longer observed along fibrils nor in the solution, and there seem to be fewer tangled networks. Final state aggregates were also imaged for samples produced under stirring (200 rpm) conditions, which show shorter fibrils as consequence of fragmentation with adsorbed vesicles (Supplementary Figure S3).

### Changes in Shape and Size of Lipid Objects Along the Aggregation Process

One clear conclusion from cryo-EM images in Figures 1–4 is that the aggregation process influences the shape and size of the lipid objects. Analyzing the cryo-EM images taken at different time points shows that the size of the vesicles dramatically decreases during the aggregation process, and that the vesicles associated with fibrils are much smaller than the initial free vesicles (Figure 5). This implies an active role of the α-syn protein in its co-assembly with lipids, as any spontaneous process in a lipid-alone sample would lead to vesicle fusion and collapse rather than vesicle fragmentation.

The comparison of the images from the four time points can provide some clues as to the sequence of events and the fate of the transient structures. For example, the irregular objects of low contrast are seen associated to fibrils in high number at *t* = 14 h, somewhat lower number at *t* = 40 h and not at all at *t* = 90 h. This implies that these objects are “consumed” during the aggregation process. At the final stage, the “blurry” and rod-like objects are replaced by small vesicles at the fibril surface. Furthermore, virtually no free lipid particles (vesicles, discs or irregular objects) are observed in the solution outside the fibrillar aggregates. The molecular events behind these changes cannot be deduced from these “still images.” However, it is possible that the disappearance of non-vesicular objects while vesicles remain may be related to difference in lipid composition between these types of objects. GM1 is an anionic lipid with large headgroup, thus promoting structures with positive curvature (Cantu et al., 2014). It is therefore likely that the observed non-vesicle structures, including micelles, as well as, rod-like and disk-like structures, are enriched in GM1, and that the relative fraction of GM1 in the vesicles is lower than the average GM1 concentration in the sample (Mojumbar et al., 2019). It has previously been shown that α-syn strongly interacts with membranes and micelles containing gangliosides and other anionic lipid and surfactants, while there is less association with zwitterionic PC membranes (Jo et al., 2000; Stockl et al., 2008; Pfefferkorn et al., 2012; Galvagnion et al., 2016; Gaspar et al., 2018; Iyer and Claessens, 2019). One can therefore expect that α-syn preferentially interacts with the non-vesicular aggregates with higher content of anionic lipids, which also explains why these are associated with fibrils during the early stages of the aggregation process. Furthermore, GM1 with a large anionic sugar headgroup has significantly higher solubility in water compared to DOPC, thus allowing for faster exchange between different self-assembled structures. Although we cannot say for sure were the lipids from these objects have gone, we can speculate that they are more strongly associated with the fibrils, possibly as a monolayer or bilayer covering the fibrillar surface (Galvagnion et al., 2019).

### Selectivity in Lipid Uptake in Fibrillar Aggregates

In order to test the hypothesis that there is an enrichment of GM1 compared to PC among the fibril-associated lipids, we analyzed the ratio of these lipid species in sedimented aggregates as well as in the supernatant over these aggregates, representing the remaining dispersed lipids that are not associated with the aggregates. The supernatant was separated from the fibrils, and these samples were freeze-dried separately. The lipids in each sample were then re-dissolved in deuterated chloroform/methanol and analyzed using one-dimensional (1D) ^1^H NMR spectroscopy. From these experiments we can detect differences in the GM1/DOPC ratios between the supernatant and the pelleted fibrils. If there is a selective uptake of one of the lipid components in the aggregates, i.e., selective depletion from solution, this will be seen as a lower ratio of this lipid species in the supernatant.

**FIGURE 6:**
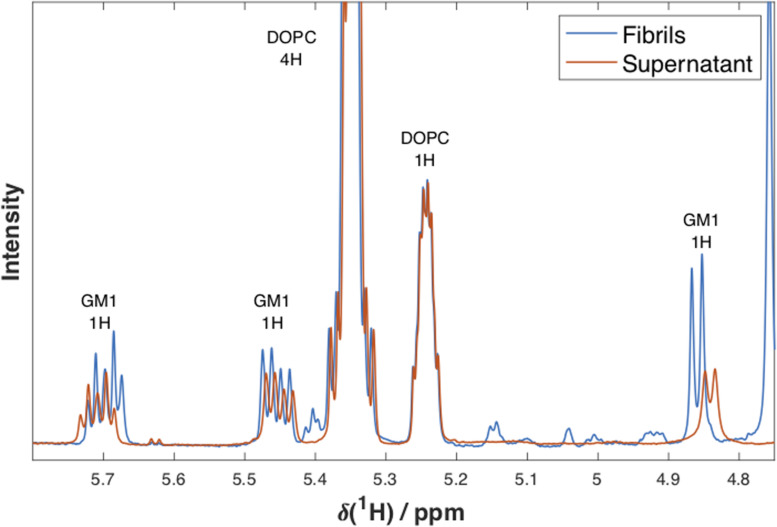
1D ^1^H NMR spectra. NMR spectra at 25°C for supernatant (red) and fibril (blue) samples formed when α-syn has aggregated in the presence of dispersed lipids DOPC/GM1 (molar ratio 7/3) at a lipid/protein molar ratio of 6/1. Peak assignments and the number of protons the peak corresponds to are indicated above each peak. The spectra were normalized with respect to the integral of the DOPC peak at 5.24 ppm. The small differences in chemical shifts observed for GM1 are most probably due to different concentrations and sample compositions of the two samples.

Experiments were performed on samples prepared with lipid dispersions with a molar ratio of DOPC/GM1 of 7/3 and L/P molar ratios of 6/1 and 10/1 (Figure 6 and Supplementary Figure S4). For both conditions, it was confirmed that lipids are present in the fibrillar samples. Figure 6 shows 1D ^1^H NMR spectra with peak assignments for supernatant and fibril samples formed when α-syn has aggregated in the presence of dispersed lipids at a L/P ratio of 6/1. The spectra are normalized with respect to the integral of the DOPC peak at 5.24 ppm. We estimate the GM1/DOPC ratio from the relative integrals of the peaks corresponding to the GM1 headgroup at ∼4.85, 5.45, and 5.70 ppm, and the peaks from DOPC at 5.24 and 5.35 ppm. For the sample with L/P ratio 6/1 shown in Figure 6, it is shown that the GM1/PC ratio is lower in the supernatant (0.29) as compared to the fibrils sample (0.42), thus implying selective uptake of GM1 into the fibrillar aggregates. The observed reduction in GM1 content in supernatant is smaller for the samples prepared using a L/P ratio of 10/1, as there is a larger excess of lipids in these samples (Supplementary Figure S4), and was not quantified.

It is here noted that the composition is not identical in the samples studied by means of NMR and cryo-TEM. Due to the low NMR signal from GM1 in samples containing only 10 mol% of this lipid, the NMR experiments were performed with model lipid mixtures with 30 mol% GM1, which is higher than what has been reported for biological membranes (Yu et al., 2009; Kolter, 2012; Mojumbar et al., 2019). Previous characterization of the DOPC:GM1 system have shown that for both compositions investigated (DOPC/GM1 molar ratio 9/1 and 7/3), the sample contain DOPC-rich vesicles and GM1-rich micelles (Mojumbar et al., 2019), and these different lipid mixtures are therefore expected to affect the aggregation process in qualitatively similar ways. The samples were then prepared at different L/P ratios, which are all within the concentration regime where added lipids have a strong effect on accelerating protein aggregation (L/P < 50, Supplementary Figure S1).

### Lipid Composition Dictates the Morphology of Mature Fibrillar Aggregates

**FIGURE 7:**
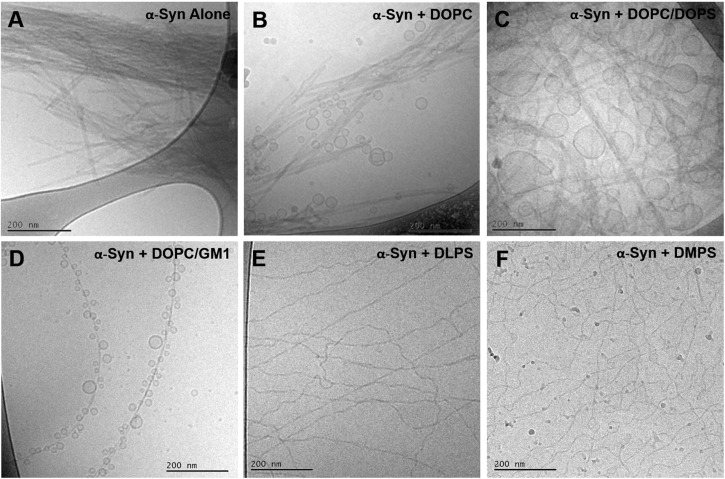
Collection of cryo-EM images of monomeric α-syn in the absence and presence of lipid vesicles with different compositions. **(A)** α-syn alone, **(B)**α-syn in the presence of pure DOPC and **(C)** DOPC/DOPS 7/3 at a L/P molar ratios of 3.6/1 (Hellstrand et al., 2013), **(D)** α-syn in the presence of pure DOPC/GM1 9/1 at a L/P molar ratio of 25/1, **(E)** α-syn in the presence of pure DLPS and **(F)** pure DMPS at L/P molar ratios of 4/1 and 2/1, respectively (Galvagnion et al., 2019). The experiments shown in panels **(A–D)** were conducted at pH 5.5, while for panels **(E,F)** at pH 6.5.

The present data clearly demonstrates lipid-protein co-assembly in systems composed of α-syn and DOPC/GM1 lipid mixtures. Lipid-protein co-assembly has previously also been demonstrated for α-syn and other lipid mixtures containing anionic lipids (Hellstrand et al., 2013; Galvagnion et al., 2019), while there is no co-assembly observed for α-syn and purely zwitterionic lipids (Hellstrand et al., 2013). With this background, we raise the question of how the lipid composition influences the structure of the fibrillar aggregates. Figure 7 shows a collection of cryo-EM images of α-syn alone and α-syn aggregated in the presence of lipid vesicles with various compositions. An immediate observation, are the notable differences between all samples. For α-syn alone there are closely packed mature fibrillar structures, the majority of which appear composed of two protofibrils intertwined (Hellstrand et al., 2013). With the addiction of lipid vesicles the morphology of such structures vary distinctively and are clearly dictated by the lipid composition. In the presence of zwitterionic DOPC vesicles, α-syn fibrils remain bundled and lipid vesicles appear randomly distributed (Hellstrand et al., 2013). For anionic DOPC/DOPS (1,2-dioleoyl-*sn*-glycero-3-phospho-L-serine) system, on the other hand, vesicles of various sizes were shown to wet the mature fibrils (Hellstrand et al., 2013). With DOPC/GM1 vesicles, thin isolated fibrils are formed heavily decorated by small vesicles of very similar sizes. In addition, these vesicles appear to be at similar distances decorating the mature fibrils. Finally for the highly charged DLPS (1,2-dilauroyl-*sn*-glycero-3-phopho-L-serine) and DMPS (1,2-dimyristoyl-*sn*-glycero-3-phospho-L-serine) systems, no vesicles are visible, and the lipids are likely strongly associated with the isolated thin and curly fibril structures (Galvagnion et al., 2019). The observed differences in fibril morphology observed for these different but related systems indicate differences in the lipid-protein interactions at different stages of the aggregation process.

## Conclusion

Lipid-protein co-aggregation leads to changes in composition, molecular organization, morphology, lipid molecular dynamics and surface properties of amyloid fibrils. It may also influence the association of amyloid fibrils with other molecules and assemblies, for example, cell membranes. We show here distinct changes in the morphology of end-stage aggregates formed in mixtures of protein and ganglioside-containing lipid vesicles compared to those of protein alone. Moreover, distinct transient species are observed during an ongoing co-aggregation process; the absence of these species at the start and end of the process implies that our cryo-EM analysis can capture a non-equilibrium situation. Using quantitative NMR analyses, we can further infer selective uptake of ganglioside lipids into the lipid-protein co-aggregates. The results have implications for the function as well as dysfunction of α-syn. Our results provide hints as to how α-syn might remodel membranes and form co-aggregates with membrane lipids. Protein-lipid co-aggregates will have distinct physico-chemical properties compared to protein-alone aggregates and extraction of components from the lipid membrane may affect the membrane properties.

## Data Availability Statement

The original contributions presented in the study are included in the article/Supplementary Material, further inquiries can be directed to the corresponding author/s.

## Author Contributions

RG, II, ES, and SL conceived and designed study. RG and II conducted all the experiments with help with the NMR experiments from GC. RG, ES, and SL wrote the article with input from II and GC. All authors contributed to the manuscript and approved the submitted version.

## Conflict of Interest

The authors declare that the research was conducted in the absence of any commercial or financial relationships that could be construed as a potential conflict of interest.
