# Supplementary material for: Transient Lipid-Protein Structures and Selective Ganglioside Uptake During α-Synuclein-Lipid Co-aggregation
Source: Front Cell Dev Biol. 2021 Feb 18;9:622764. doi: 10.3389/fcell.2021.622764 (PMC7930334; doi:10.3389/fcell.2021.622764)
Supplement: Supplementary file 1 [file Data_Sheet_1.PDF]

Supporting Information

ThT aggregation kinetics of α-syn in the presence of ganglioside GM1 containing lipid membranes

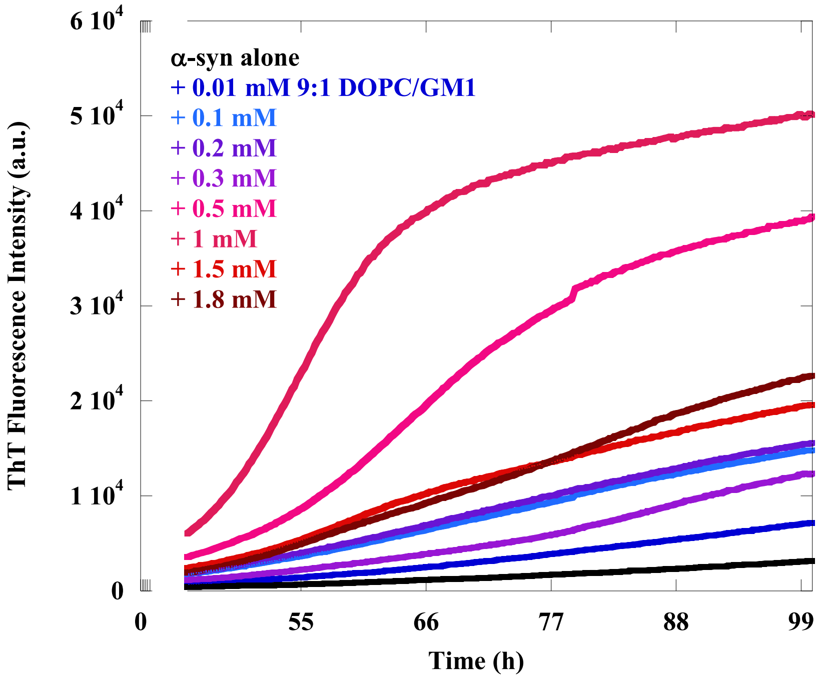

Figure SI1. ThT aggregation kinetics of α-syn in the presence of ganglioside GM1 containing lipid membranes. The aggregation kinetics of 20 μM of α-syn was monitored in the presence of 9:1 DOPC/GM1 lipid vesicles with concentrations ranging from 0.01–1.8 mM (L/P ratios 0.5–90), in nonbinding PEGylated plates under quiescent conditions at 37ºC in 10 mM MES pH 5.5 buffer. The average traces of at least 3 experimental replicates are represented as solid lines.

Adsorption of α-syn to negatively charged DOPC/GM1 supported lipid bilayers

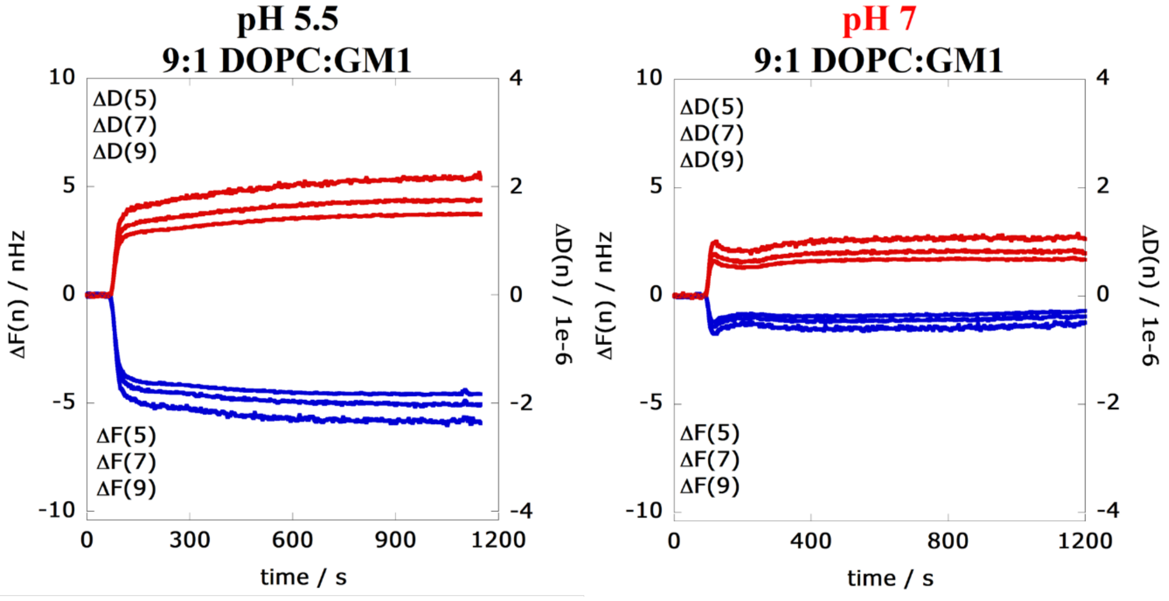

Figure SI2. Adsorption of α-syn to negatively charged DOPC/GM1 (molar ratio 9/1) supported lipid bilayers at SiO_2 coated quartz crystals. Experiments were performed in 10 mM MES buffer,

pH 5.5 and in 10 mM HEPES pH 7. Shifts in frequency (blue) and dissipation (red) were monitored with QCM-D after injecting 4 μM α-syn to the solution that is in contact with the supported lipid bilayers. Each experimental condition was repeated at least three times.

Imaging the final stage aggregates formed under agitation

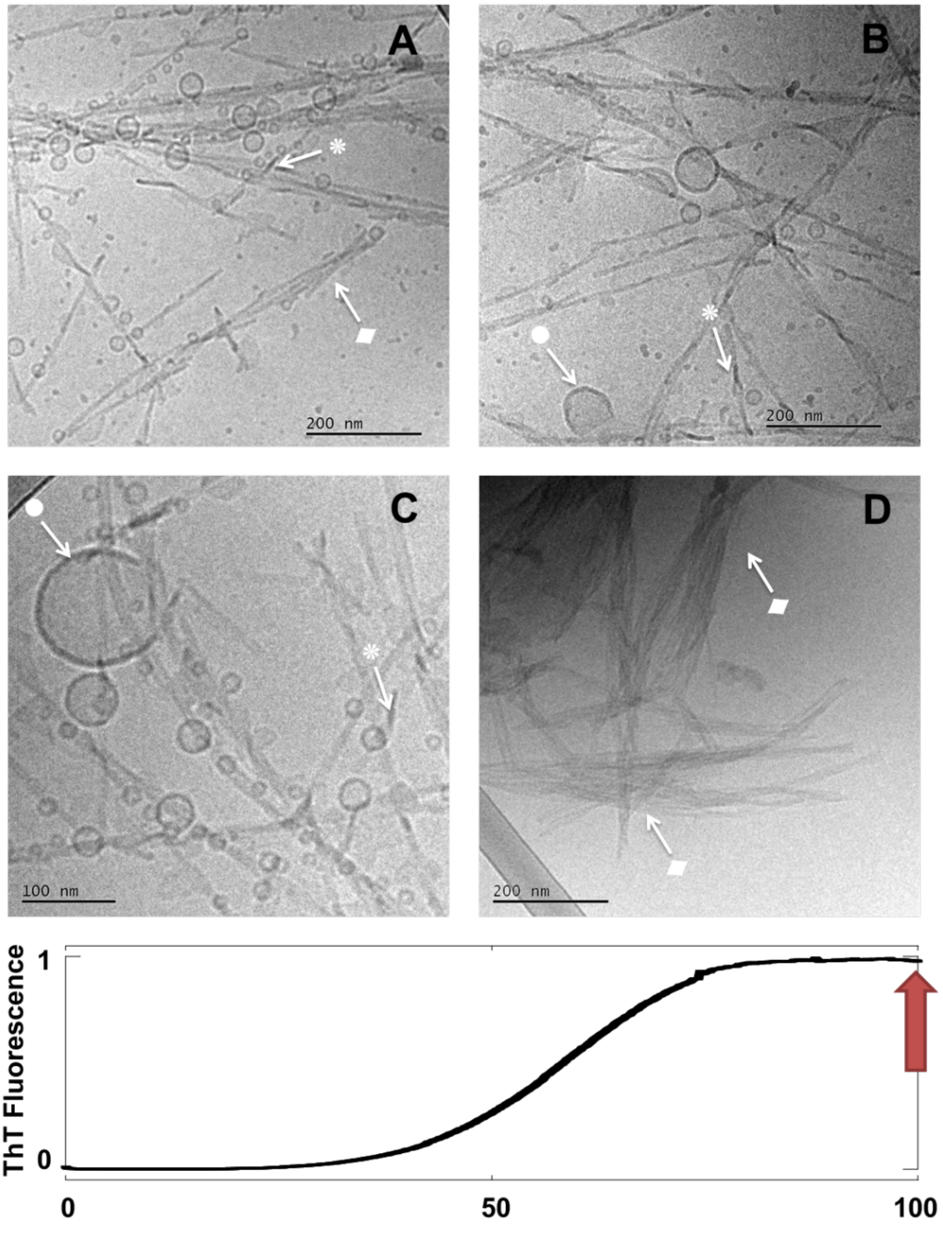

Figure SI3. **Imaging the final stage of aggregated α-syn alone and in the presence of lipids under shaking conditions**. The conditions were the same as in Figure 4, except that here the aggregation reaction was performed under shaking conditions (200 rpm). Arrows with ● symbols indicate examples of lipid vesicles, ♦ fibrillar structures and ✳ point to lipid disc-like objects. The top panels and lower left panel correspond to samples of α-syn aggregated in the presence of lipids under shaking conditions. The lower right panel corresponds to α-syn alone aggregated under shaking conditions. This comparison enables us to visualize changes in morphology of the aggregates formed in mixtures of protein and lipid vesicles compared to those of protein alone. The main difference for the lipid-protein samples prepared under quiescent (Figure 4) and stirring conditions resides in that the agitated fibrils are shorter, which is in line with the dominant fragmentation process associated with stirring. Again,

adsorbed lipid vesicles are of vast majority and of smaller sizes compared to the vesicles at t = 0, although some large vesicles are also seen.

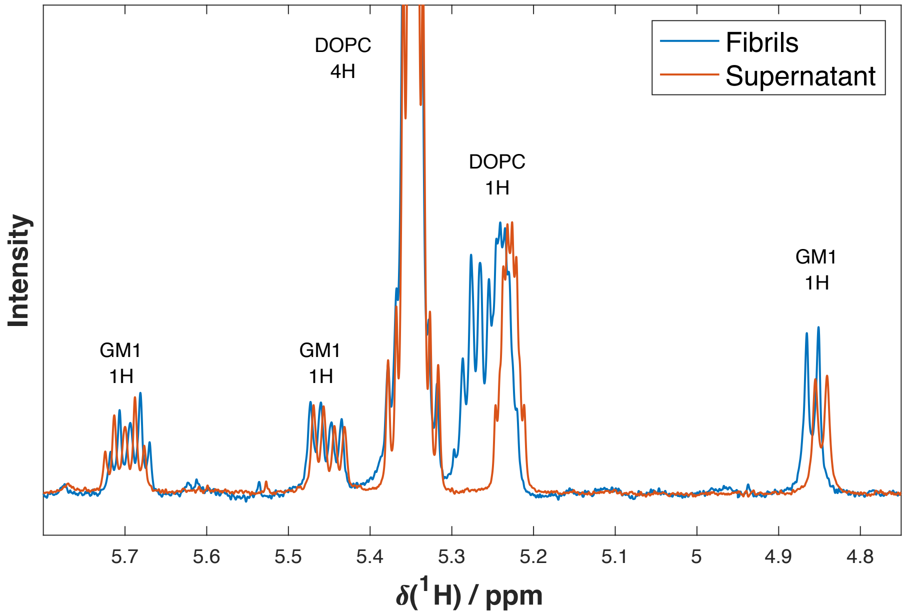

Figure SI4. 1D ^{1}H NMR spectra. NMR spectra at 25 °C for supernatant (red) and fibril (blue) samples formed when α-syn aggregated in the presence of dispersed lipids DOPC/GM1 (molar ratio 7/3) at a lipid/protein molar ratio 10/1. Peak assignments and the number of ^{1}H the peak corresponds to are indicated above the peaks. The spectra were normalized with respect to the integral of the DOPC peak at 5.35 ppm.

Supplementary Method

Quartz Crystal Microbalance – Dissipation Measurements

A Q sense E4 instrument (Gothenburg, Sweden) and quartz crystals covered by a thin gold film coated with 50 nm SiO_2 (QSX 303, Q-sense) were used to perform the QCM-D measurements. The crystals had a fundamental frequency of 4.95 MHz. The experimental temperature was 25°C. The quartz crystals were stored in 2% SDS solution. The crystals before use were rinsed with deionized water and ethanol, dried with nitrogen and finally treated in a plasma cleaner (model PDC-3XG, Harrick Scientific Corp., Pleasantville, NY) under reduced air pressure for 5 minutes. A peristaltic pump (Ismatec IPC-N4) controlled the flow through in the four measuring cells. After placing the crystals in the instrument, stable baselines for the frequency and dissipation were achieved flowing water through the cells. The vesicle dispersions in aqueous solution with 100 mM NaCl were passed through the measuring cells at a flow rate of 100 µl/min for 10 minutes. After this period, the NaCl aqueous solution was replaced with water and then equilibrated with the desired experimental buffer (10 mM MES pH 5.5 or 10 mM HEPES pH 7) prior to protein injection. The protein was diluted to a concentration of 4 µM, injected at a flow rate of 50 µL/min. For most of the experiments the total time of protein injection was approximately 20 min.
